# Supplementary material for: Conditional chemoconnectomics (cCCTomics) as a strategy for efficient and conditional targeting of chemical transmission
Source: eLife. 2024 Apr 30;12:RP91927. doi: 10.7554/eLife.91927 (PMC11060718; doi:10.7554/eLife.91927)
Supplement: Figure 3—source data 3. [file elife-91927-fig3-data3.zip › Figure 3 source data3 part 1.zip!/Figure 3-Source data 3-part1/1067-9-CRISPResso_Report_c33kf3u3.zip!/CRISPResso_on_1067_10/9.Alleles_frequency_table_around_sgRNA_agccaggtgtcgcagacgat.pdf]

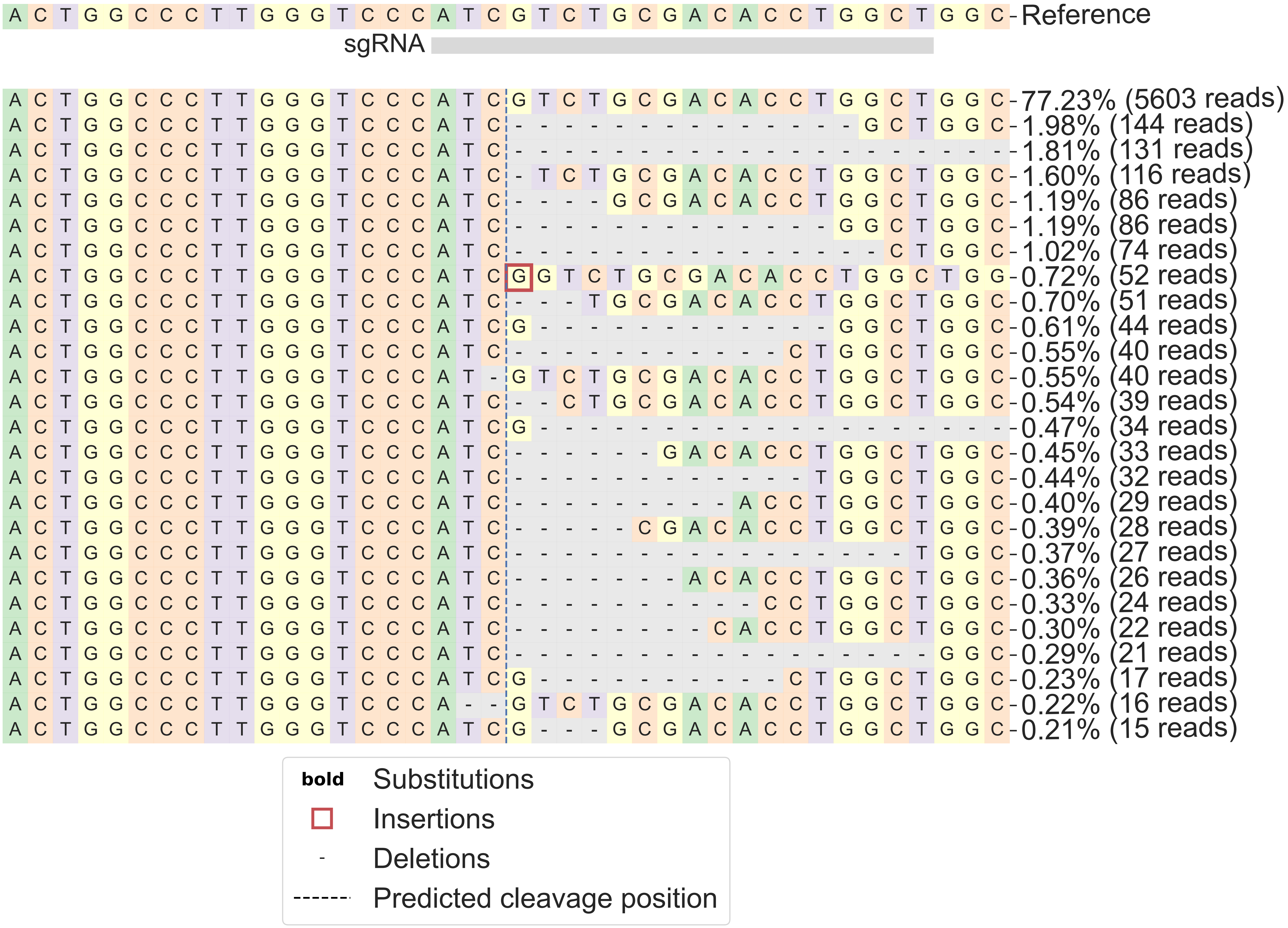

Reference
sgRNA
77.23% (5603 reads)
1.98% (144 reads)
1.81% (131 reads)
1.60% (116 reads)
1.19% (86 reads)
1.19% (86 reads)
1.02% (74 reads)
0.72% (52 reads)
0.70% (51 reads)
0.61% (44 reads)
0.55% (40 reads)
0.55% (40 reads)
0.54% (39 reads)
0.47% (34 reads)
0.45% (33 reads)
0.44% (32 reads)
0.40% (29 reads)
0.39% (28 reads)
0.37% (27 reads)
0.36% (26 reads)
0.33% (24 reads)
0.30% (22 reads)
0.29% (21 reads)
0.23% (17 reads)
0.22% (16 reads)
0.21% (15 reads)
bold Substitutions
Insertions
Deletions
Predicted cleavage position
